# Comparative transcriptome analysis of *Labeo calbasu* (Hamilton, 1822) from polluted and non-polluted rivers in India

**DOI:** 10.1371/journal.pone.0320358

**Published:** 2025-04-10

**Authors:** Smruti Priyambada Pradhan, Hirak Jyoti Chakraborty, Ayushman Gadnayak, Subhashree Subhasmita Raut, Dhruba Jyoti Sarkar, Anu Sharma, Dwijesh Chandra Mishra, Mohammad Samir Farooqi, Bijaya Kumar Behera, Basanta Kumar Das, for the Centre for Agricultural Bioinformatics (CABin) Scheme, ICAR-IASRI, New Delhi

**Affiliations:** 1 ICAR-Central Inland Fisheries Research Institute, Kolkata, India; 2 ICAR-Indian Agricultural Statistics Research Institute, New Delhi, India; Bangladesh Agricultural University, BANGLADESH

## Abstract

*Labeo calbasu* (*L. calbasu*) is an important detrivore fish in an ecosystem. So, the present transcriptome study was undertaken in relation to polluted and non-polluted water sources from a natural perennial river system. The Illumina NovaSeq 6000 platform was used to perform transcriptome analysis on liver samples of *L. calbasu* that were collected from the Ganga and Yamuna rivers. From 8744 differentially expressed genes (DEGs), 2538 were upregulated, and 6206 were downregulated in response to pollution stress. Biologic process (BP), cellular component (CC), molecular function (MF), and Gene Ontology (GO) demonstrated that relevant genes were associated with peptide metabolic process, cytosol, RNA binding, etc. In the Kyoto Encyclopedia of Genes and Genome (KEGG) analysis, ribosomal and metabolic pathways were more important due to the high False discovery rate (FDR) and the involvement of many genes. Transcripts of uncertain coding potential (TUCP) and various RNAs like mRNAs and long noncoding RNAs (lncRNAs) orchestrate fish cellular responses to environmental stressors in polluted waters, where aquatic ecosystems are threatened. FGG mRNA is co-expressed in both up and down-regulation in the liver of *L. calbasu*. In conclusion, *L. calbasu* collected from the Yamuna River have highly pollution-induced ribosomal pathways involving genes like Rpl19, rpl23Ae, rps2e, rps10e, rps15e, and rps7e, etc, which is important for pollution biomarker study. RANBP2 and egr1 lncRNA are the most significantly interlinked with ndc1 and fosab lncRNA.

## Background

Fish, in particular, are exposed to extremely hazardous environments from an early age. Because of alterations in morphology, biochemistry, physiology, and chemicals, they react to environmental pollutants in a variety of ways [1]. *Labeo calbasu*, also known as “black rohu,” is a detritivorous fish widely spread in open water bodies like lakes, rivers, reservoirs, and wetlands. It forms significant fisheries in the Indian subcontinents. As it is an indigenous species, it is commercially important and may be a component of a system of polycultures. It is also responsible for various aquafarming practices and is widely acceptable for consumption in larger communities (Ganga, Yamuna, Godavari, Narmada, Ken, Tons, and Paisuni). Consumers are more inclined to purchase this species due to its massive dimensions. As a bottom feeder, it consumes deceased and decaying matter thereby serving as a scavenger and enhancing the sanitation of lakes, ponds, reservoirs, and rivers [2]. Being detritivorous in feeding habits, the species is included in the National River Ranching programme of river Ganga, which covers the lower and middle stretches of the river to make a “Nirmal Ganga” (clean Ganga) [3]. As fish is used as a cleaner for sewage control and human consumption, it is essential to understand the molecular and biological processes responsible for its growth, metabolism, and cellular function. During exposure to various biotic and abiotic stresses, there is a comprehensive molecular mechanism underlying this fish in its natural habitats [4,5].

Supplying water for industrial, agricultural, and daily needs, rivers are among the most vital sources of potable water for human consumption. The movement of numerous items, including trash, pollutants, and waste, is aided by the flow of river systems. Additionally, this responsibility is borne by the rivers that replenish or maintain the groundwater aquifers [6]. Currently, rivers are burdened with tremendous loads due to anthropogenic activities such as the discharge of municipal refuse, industrial operations, and riverbed mining. Consequently, the water quality of rivers has significantly declined, posing threats to aquatic life and human existence [7].

Many environmental and human health complications ensue from releasing numerous contaminants into water [8]. The aforementioned activities gradually deteriorate the aquatic ecosystem, affect fish health, and reduce water purity, presenting substantial environmental challenges for the nation. Over the past several decades, the Yamuna has been regarded as one of India’s most polluted rivers [9]. Various sectors, including pulp and paper, sugar, tanneries, steel factories, thermoelectric power plants, food processing facilities, rubber, textiles and clothing, leather products, chemicals, pharmaceuticals, oil refineries, and glass predominantly cause the degradation of the river Yamuna [10]. Domestic sources and sludge from industries, mainly heavy metals like Cd, As, Cr, Fe, and Zn, are mixed for approximately 9.63 kilometers annually in the Yamuna River basin [11].

Having this, the present study is focused on a transcriptomic analysis covering gene function and analysis of crucial pathways in habitat from a mostly polluted and nonpolluted river. The fish liver is responsible for excretion, secretion, and regulating metabolism and immunity. It is the gold standard for nutritional metabolism research because of its central role in metabolizing carbohydrates, proteins, and lipids [12]. Further, the liver expresses several genes via multiple pathways, and it would be impractical to study the impact of different pollutants on metabolic pathways, mainly focusing on proteins. Transcriptome analysis using high-throughput sequencing has dramatically advanced our knowledge of the pathway analysis of a few specific genes that are upregulated, downregulated, and coexpressed in fish [13]. It can employ RNA sequencing, also called RNA-seq, to analyze gene expression across the whole genome. By examining the functional genome and the temporal expression of its components in cells and tissues, transcriptome profiling aims to explore variations in gene expression across many organs, tissues, cells, and stages of life in the organism of interest [14]. While studying transcriptome data in fish, RNA-seq has shown to be an invaluable tool for detecting susceptible and particular candidate genes [15]. Since the liver is the most important organ that indicates the general health of fish, studying alterations in its transcriptomes might shed light on the mechanisms and pathways of stress response. The liver removes many kinds of pollutants, poisons, and toxins from the body [16]. Generally, the liver transcriptome is an important area in pollution research, which gives indications of the xenobiotic implications and its regulatory pathway. Researchers found many promising indicators to understand the transcriptomes of livers under pollution stress. A comparative transcriptome study on the liver of *L. calbasu* from two rivers (Yamuna and Ganga) using the Illumina NovaSeq 6000 platform was conducted to examine the impacts of pollutants on fish in the riverine environment.

## Methods

### Ethical approval

The current investigation, sample collection, experimental design, and fish sacrifice all adhered to Indian ethical standards approved by the Institute Animal Ethical Committee (IAEC) (Number. CIFRI/IAEC-2021-2022/04). Tricaine (Ethyl 3-aminobenzoate methanesulfonate, MS-222 Sigma-Aldrich, USA) was used as anesthesia and all efforts were made to minimize fish suffering.

### Sample collection and physicochemical assessments

Each individual of L. calbasu was collected from polluted sites of the river Yamuna at New Delhi (as treated) (28034’30“N & 77033’30” E) and pristine sites of the river Ganga at Barrackpore (as control) (22050’0”N & 88020’0” E) on April 2021. There were 1-2 fishes (Average weight 450gm) pulled on sampling at the river Yamuna Ghat, New Delhi, and 1-3 fishes (Average weight 400gm) at the river Ganga at Barrackpore. The fish have no visible signs of clinical symptoms and are healthy. At first, the anesthetic agent (Tricaine solution) was freshly prepared by dissolving it in distilled water to obtain a concentration of 1mg/L. Then, the fish were immersed in it and kept for 5 minutes till they became subconscious. Therefore, the fish were sacrificed, and the liver sample was collected aseptically [17]. Immediately after collection, the liver samples were transferred to RNAlater and kept at -80 °C until RNA extraction. To evaluate and compare the physicochemical characteristics and habitat quality, water and sediment samples were taken from both sites. The water quality parameters, e.g., indicators, pH, dissolved oxygen (DO) (ppm), biological oxygen demand (BOD) (ppm), specific conductivity (μS/cm), salinity (ppt), and chemical oxygen demand (COD) (ppm) were analyzed. The sediment characteristics, e.g., pH, specific conductivity, total nitrogen, available phosphate, and organic carbon, were also analyzed.

### RNA extraction, library construction, and sequencing

Trizol reagent (Invitrogen, California, USA) was used for RNA extraction in accordance with the manufacturer’s instructions. RNA concentration and integrity of the sample were measured on an Agilent 2100 Bioanalyzer (Agilent Technologies) using an RNA Nano Bioanalysis chip. Utilizing Truseq mRNA kits and the Illumina HiSeq 2000 equipment with 100 bp paired-end (PE) reads, Macrogen Inc. (Seoul, Korea) carried out the mitochondrial RNA enrichment, library preparation, and sequencing [18]. Samples with an RNA Integrity Number (RIN) greater than 6.0 were used for RNA library construction. The sequencing library was constructed with the Illumina NovaSeq 6000 instrument, producing 100 bp paired-end (PE) reads. In the NCBI database, the generated *L.calbasu* liver samples were deposited under the submission numbers SRR22956457 and SRR22956458.

### Quality control

The data was preprocessed using Fastp (https://github.com/OpenGene/fastp) [fastp: an ultra-fast all-in-one FASTQ preprocessor] v.0.20.1 (parameters: -f 10 -q 30 -l 50 -c --adapter_fasta). Raw data assessment was performed using FastQC v.0.11.9 [FastQC: a quality control tool for high throughput sequence data] (default parameters).

### Transcriptome analysis

The cleaned reads were considered for de novo assembly using Trinity v2.11.0 with default parameters. Transcripts with coding potential were superscaffolded using TransDecoder (default parameters). The set of transcripts generated from TransDecoder was quantified using the align_and_estimate_abundance.pl script with the RSEM v2.14.0 method. The resulting abundance counts were imported into the edgeR exact Test for differential expression analysis (parameters: dispersion =  0.1). Differentially expressed genes (DEGs) were identified based on the following criteria: A log2FoldChange threshold of ≥  2.0, Statistical significance controlled using an adjusted p-value (FDR ≤  0.05) to minimize false positives. Precomputed eggNOG-based orthology assignments was used for functional annotation by eggNOG Mapper (Version of Database5.0.2). Significant DEGs were subjected to Gene Ontology (GO) enrichment and pathway analysis using an R package with a False Discovery Rate (FDR) cut-off of 0.05, including the background database of *Danio Rerio*. Types of RNAs, including mRNAs and long non-coding RNAs (lncRNAs), were classified and analysed to understand their functional roles in response to pollution stress. lncRNAs were identified based on standard criteria, including transcript length (>200 bp) and lack of coding potential.

## Results

### Physicochemical differences among sampling sites

**Fig 1:**
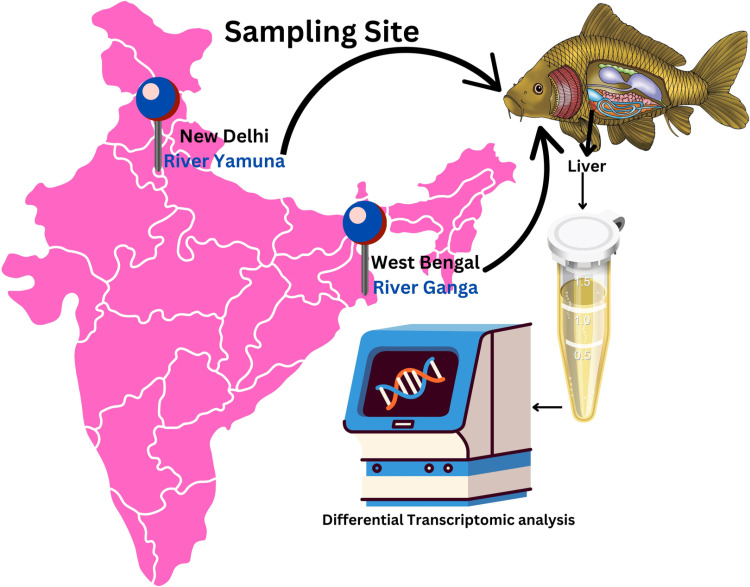
Geographical Map Showing the water and sediment sampling sites. Water and sediments were collected from the river Ganga at Daspara Ghat, Barrackpore (as a non-polluted site), and Yamuna Ghat, New Delhi (as a polluted site), which was generated by Canva Pro (https://www.canva.com/en_in/pro/).

Two sampling sites were chosen for this study: one site defined as non-polluted, i.e., Barrackpore, the Ganga River, and the other one defined as polluted, i.e., New Delhi, the Yamuna River Fig 1. At both of these locations, samples of water and sediment were taken. The physicochemical characteristics of the water from both locations, such as pH, dissolved oxygen (DO) (ppm), biological oxygen demand (BOD) (ppm), specific conductivity (μS/cm), salinity (ppt), and chemical oxygen demand (COD) (ppm), are measured to compare the impact of pollution on aquatic life. Table 1 presents the physicochemical characteristics of water and sediments obtained from New Delhi, Yamuna River (polluted), and Barrackpore, Ganga River (non-polluted).

**Table 1:** Comprehensive Summary of Physiochemical parameters of water and sediment samples collected from different sites of the river Ganga (non-polluted) and Yamuna (polluted).

Samples	Parameters	Ganga (Non-polluted)	Yamuna (Polluted)
Water	Dissolved oxygen concentration (DO) (ppm)	5.60	4.5
	pH	7.60	7.41
	Specific conductivity (µs/cm)	375	633
	Salinity (%)	0.40	0.29
	Biochemical oxygen demand (BOD) (ppm)	1.4	7.6
	Chemical oxygen demand (COD) (ppm)	5.0	8.5
Sediment	pH	7.90	8.3
	Specific conductivity (µs/cm)	254	866
	Total nitrogen (%)	0.04	0.08
	Available phosphate (mg/100g)	2.0	2.9
	Organic c (%)	0.30	0.45

Barrackpore and New Delhi water and sediment pH values were 7.60, 7.41, and 7.90, 8.3, respectively. The conductivity values of Barrackpore and New Delhi water samples were 375 and 633 µ S/cm. The quantity of dissolved salts in water is measured as salinity. The water salinity of this study was 0.40 and 0.29 ppm for Barrackpore and New Delhi. The water DO shows a lower concentration of 5.60 ppm in Daspara water compared to Yamuna ghat, New Delhi water, with 4.5 ppm concentration.

The BOD is a metric for expressing the concentration of organic molecules in a sample as a function of the oxygen needed for aerobic microbial metabolism. The BOD of Barrackpore and New Delhi water samples were 1.4 ppm and 7.6 ppm. The concentration of BOD in a river is influenced significantly by the quantity of low-volatile matter discharged in coal refuse, which is represented by the COD value. The COD values of the Barrackpore and New Delhi water samples were 5.0 ppm and 8.5 ppm. Sediment conductivity measurements at Barrackpore were 254 μS/cm, whereas at New Delhi, the values were 866 μS/cm.

### Transcriptome sequencing, assembly, and alignment

We constructed and sequenced two libraries using Illumina NovaSeq 6000 sequencing technology: one from an unpolluted section of the Ganga River and one from a contaminated section of the Yamuna River. This will shed light on how contamination affects the patterns of mRNA expression in the liver of *L. calbasu*. The sequencing produced 104.547M and 82.038M paired-end-raw-reads for the liver of *L. calbasu* from polluted and non-polluted areas. A total of 97.854G and 75.83G clean reads were retrieved from polluted and pristine sites Table 2. The clean readings were positioned using the Trinity tool relative to the *L. calbasu* De novo assembly.

**Table 2:** Comprehensive Summary of sequence reads of *L. calbasu* from non-polluted sites of the river Ganga and polluted sites of the river Yamuna.

Sample	Raw reads	Total bases	Clean reads	GC Content (%)
Barrackpore	82.038M	13.208G	75.83G	60
New Delhi	104.547M	16.832G	97.854G	56

**Fig 2:**
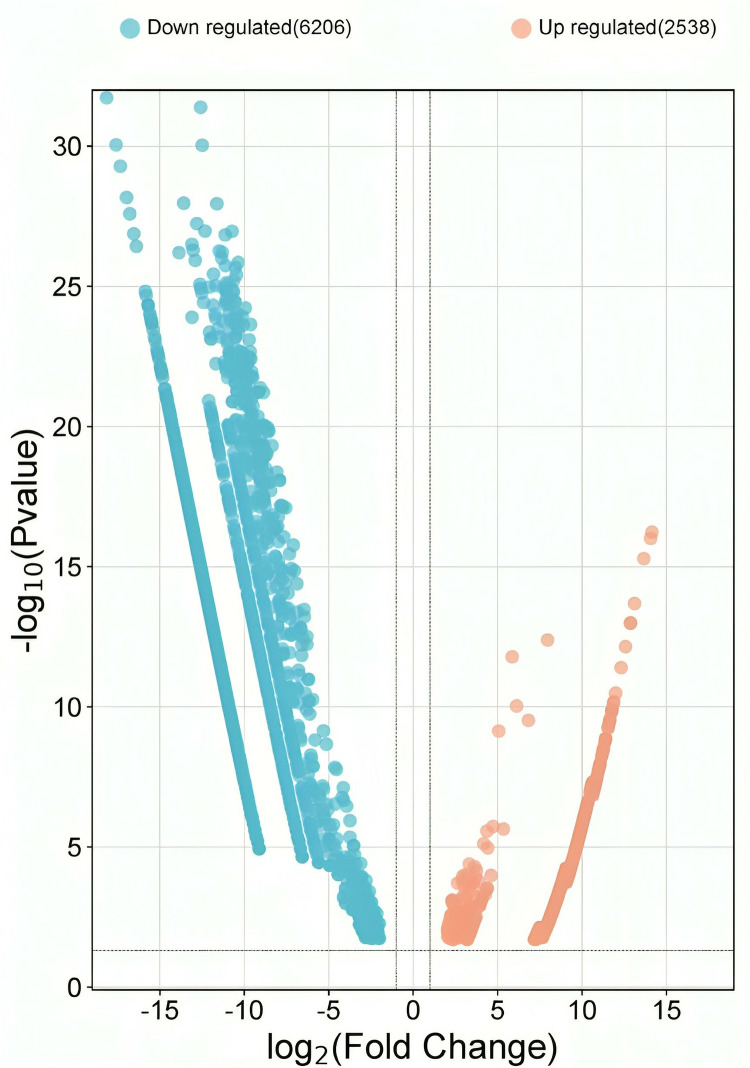
Volcano Plot of Differential Gene Expression Between Pristine and Contaminated Groups. The volcano plot illustrates gene expression differences between the pristine and contaminated groups. Each point represents a gene, with red and blue dots indicating significantly upregulated and downregulated genes, respectively. The x-axis represents the log-fold change in gene expression between the two groups, while the y-axis represents the statistical significance (−log10 of the FDR-adjusted P-value). Genes with an FDR-adjusted P-value <  0.05 are considered significantly differentially expressed (DEGs), highlighting key genetic variations associated with contamination.

Liver tissue from polluted and non-polluted sites of *L. calbasu* was compared to study the overall gene expression response. In comparison to the non-polluted sites, the polluted site *L. calbasu* liver exhibited 2538 upregulated genes and 6206 downregulated genes (FDR-adjusted P-value < 0.05 and | Log2(fold change) |  >  1 by using t-test statistical analysis (Fig 2).

### Gene ontology analysis

**Fig 3:**
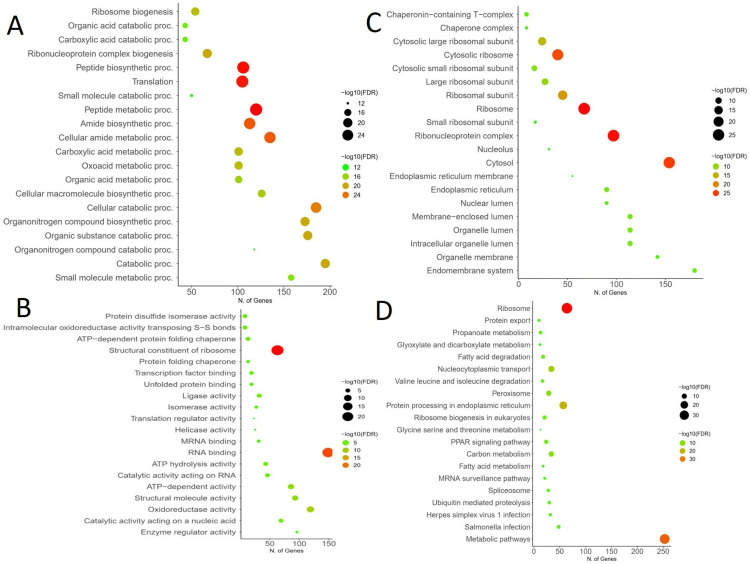
The GO analysis was conducted using a p-value of less than 0.05, and it was categorized according to (A) biological process (BP), (B) cellular component (CC), and (C) molecular function (MF). The large red circle represents the highly significant enriched genes, and the other coloured circles are also significantly enriched according to the node size analyzed through the R package. The (D) KEGG pathways for DEGs were analyzed through the R package based on a p-value of < 0.05. The large red circle represents the highly significant enriched genes, and the other coloured circles are also significantly enriched according to the node size.

Using GO, we could classify genes according to their functions; this improved our comprehension of the biological significance of specific genes exposed to riverine contamination. An enriched p-value of less than 0.05 was used to categorize the genes into three levels: biological process, cellular component, and molecular function after they were annotated using GO. In the BP group, most unigenes were linked to ribosome biogenesis, peptide biosynthetic process, peptide metabolic process, translation, cellular amide metabolic process, amide biosynthesis process, cellular catalytic process, ribonucleoprotein complex biogenesis, carboxylic acid metabolic process, organonitrogen compound biosynthetic process, oxoacid metabolic process, organic substance catabolic process, and catabolic process. In the CC, unigenes were linked with ribosome, ribonucleoprotein complex, cytosolic ribosome, cytosol, ribosomal subunit, cytosolic large ribosomal subunit, cytosolic small ribosomal subunit, large ribosomal subunit, small ribosomal subunit, chaperonin-containing T-complex, endoplasmic reticulum, membrane-enclosed lumen, organelle lumen, and intracellular organelle lumen. In MF, unigenes were linked with a structural constituent of ribosome, oxidoreductase activity, RNA binding, ATP-dependent activity, and structural molecule activity (Fig 3).

In BP, the peptide biosynthetic process, translation, cellular amide metabolic process, amide biosynthesis process, and peptide metabolic process were most prevalent in response to high FDR value and cellular catalytic process, catabolic process, organonitrogen compound biosynthetic process, and organic substance catabolic process were most prevalent in response to large number of genes involvement. In the CC, cytosolic ribosome, ribosome, ribonucleoprotein complex, and cytosol were most prevalent in response to high FDR value and endomembrane system, organelle lumen, organelle membrane, membrane-enclosed lumen, and intracellular organelle lumen were most relevant in response to large number of genes involvement. The cytosol was the most relevant, as it has a high FDR value, and many genes were involved. In response to high FDR value, structural constituents of ribosome and RNA binding were most prevalent, and oxidoreductase activity, ATP-dependent activity, and structural molecule activity were most prevalent in response to many genes involved in MF.

### KEGG pathway analysis

KEGG pathway analysis was performed for the functional characterization. There are crucial pathways like ribosome, metabolic, nucleocytoplasmic transport, fatty acid degradation, spliceosome, protein export, and salmonella infection, shown in KEGG pathways (S1 Table). In this analysis, crucial genes involved in the ribosome and metabolic pathways have the highest FDR values, and many genes are involved in response to riverine pollution at the rivers Ganga, Barrackpore, and Yamuna, New Delhi. These pathways were more abundant in the up-regulated and down-regulated genes, respectively. Most of the genes are associated with crucial pathways of KEGG. In metabolic pathways, 253 genes are linked with an FDR value of 2.264, while 64 genes are linked with ribosome pathways (RP) with a high FDR value of 7.767 shown in Table 3.

**Table 3:** Comprehensive Summary of KEGG pathways along with their corresponding fold enrichment, Number of genes, and their enrichment score.

Si. No	Pathways	Number of Genes	Enrichment Score	Fold Enrichment
1	Ribosome	64	1.86E-39	7.767950731
2	Protein export	10	3.20E-06	7.172114
3	Propanoate metabolism	13	2.56E-07	6.616853
4	Glyoxylate and dicarboxylate metabolism	12	1.00E-05	5.25955
5	Fatty acid metabolism	18	7.99E-06	3.737049
6	Nucleocytoplasmic transport	34	3.90E-14	5.061076
7	Valine leucine and isoleucine degradation	17	8.52E-07	4.624777
8	Peroxisome	29	4.38E-11	4.622029
9	Protein processing in endoplasmic reticulum	57	3.54E-21	4.565396
10	Ribosome biogenesis in eukaryotes	21	5.42E-08	4.539064
11	Glycine serine and threonine metabolism	13	2.83E-05	4.459184
12	PPAR signaling pathway	24	3.27E-08	4.161402
13	Carbon metabolism	34	3.09E-11	4.095222
14	Fatty acid metabolism	18	7.99E-06	3.737049
15	mRNA surveillance pathway	21	6.46E-06	3.381139
16	Spliceosome	28	9.13E-07	3.089526
17	Ubiquitin mediated proteolysis	30	9.13E-07	2.958497
18	Herpes simplex virus 1 infection	32	1.90E-06	2.744113
19	Salmonella infection	48	2.18E-07	2.412022918
20	Metabolic pathways	253	5.88E-34	2.264321288

**Fig 4:**
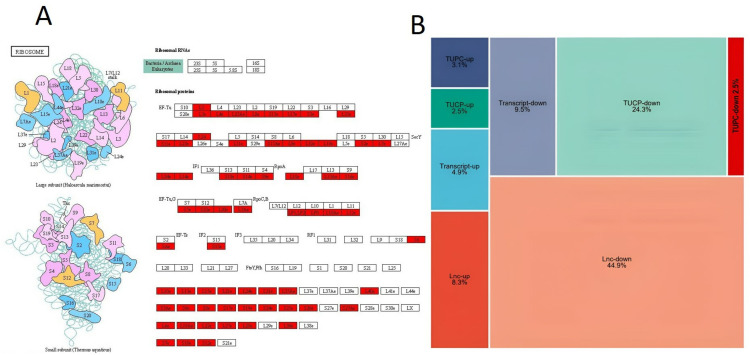
(A) Overview of ribosome pathways, illustrating key molecular interactions involved in protein synthesis. (B) Treemap visualization of different types of enriched RNAs, mapped using the KEGG pathway database, providing insights into their functional significance.

Crucial genes like mgat2, ehhadh, pik3cb, pmvk, ptdss2, mthfd2, cox11, acsbg1, and fktn are associated with metabolic pathways. Most genes, including rpl3, rpl19, rpl7, rpl22, rpl23Ae, rps2e, rps17, rps19, rps10e, rps15e, and rps7e etc, are overexpressed in the ribosome pathways (Fig 4). These ribosomal proteins may encode either a large subunit (Haloarcula marismortui) or a small subunit (Thurmus aquaticus). There are 39 rpl, and 26 rps genes are overexpressed in the *L. calbasu* genome. Protein synthesis is carried out by ribosomes, which are complex molecular mechanisms. They comprise two parts: the 40S subunit and the large 60S subunit. The building blocks of these subunits include proteins and ribosomal RNA (rRNA).

From the total number of significant genes of *L. calbasu*, 53.2% lnc RNAs are being classified, of which 44.9% lnc RNAs contribute to downregulation and 8.3% lnc RNAs contribute to upregulation. The total number of up-and down-regulated transcripts is 14.4%, up- and down-regulated transcripts of uncertain coding potential (TUCP) is 26.8%, and TUPC is 5.6% (Fig 4). Numerous studies on fish have shown that lncRNAs are also implicated in the stress response gene expression network.

**Fig 5:**
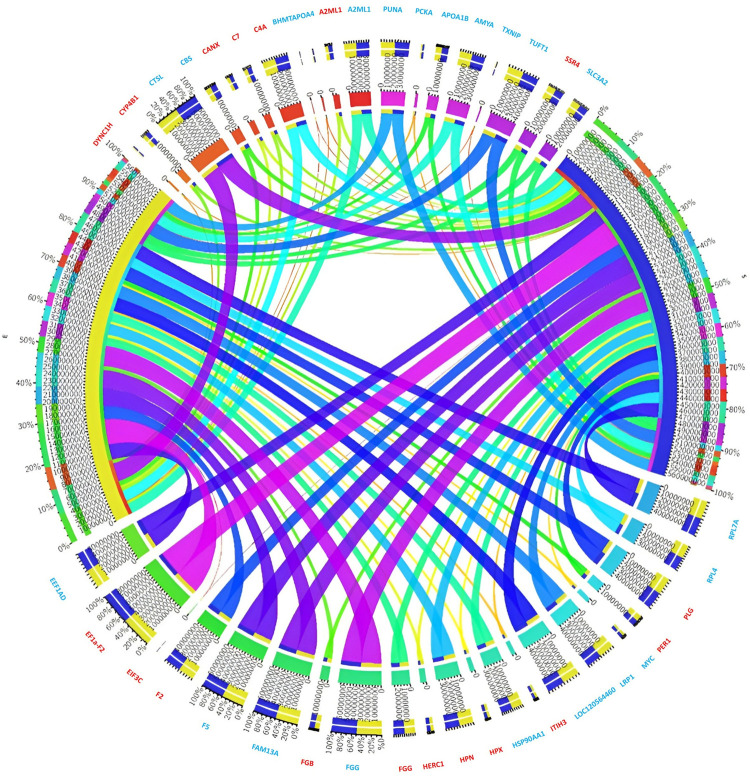
Circos plot visualization of top 20 upregulated and downregulated mRNAs of significant genes, illustrating differential gene expression patterns and their potential regulatory interactions.

Total expressed transcripts are used to determine the top 20 differentially expressed mRNAs that are either up-regulated or down-regulated, as shown in (Fig 5). The expressed up-regulated mRNAs DYNC1H, CYP4B1, CANX, C4A, BHMTAPOA4, A2ML1, SSR4, PLG, PER1, ITIH3, HPX, HPN, HERC1, FGG, FGB, EIF3C, and EF1a-F2 etc are displayed in red colour. The expressed down-regulated mRNAs CTSL, CBS, A2ML1, punA, pckA, FGG, apoa1b, amyA, TXNIP, TUFT1, SLC3a2, and RPL4, etc. are displayed in blue colour.

**Fig 6:**
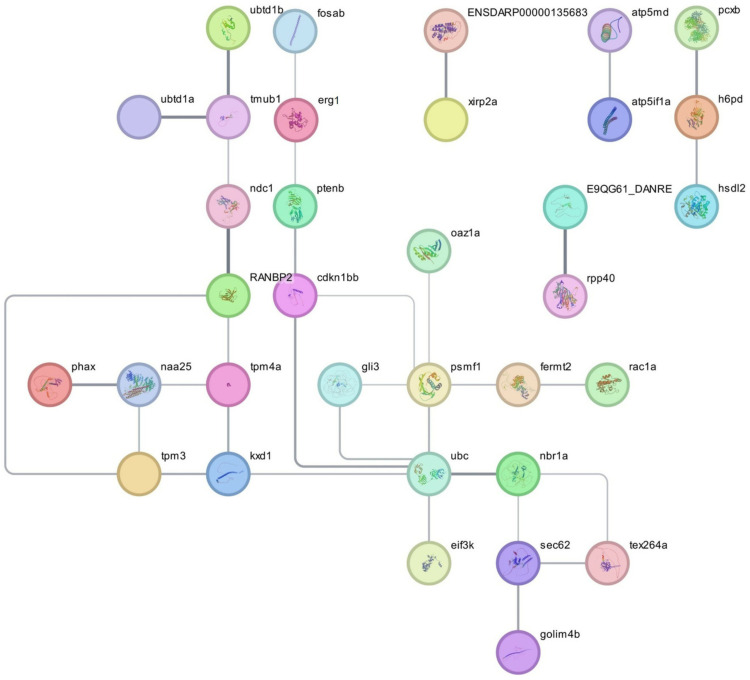
Visualization of 35 significant lncRNA interaction networks, constructed using the STRING database and analyzed through network topology in Cytoscape 3.9.1. This representation highlights key connections and interactions among lncRNAs, providing insights into their functional roles.

Thirty-three lncRNAs are interlinking, as shown in (Fig 6). Ubc (ubiquitin C) is interlinked with six lncRNAs, i.e., kxd1, nbr1a, gli3, psmf, 1ei3k, and cdkn1bb, with the functions of ubiquitin homologs, ubiquitin-like domain, PIP3 activates AKT signaling, SCF(Skp2)-mediated degradation of p27/p21, Cellular responses to stress, Cellular Senescence, PCP/CE pathway, GLI3 is processed to GLI3R by the proteasome, Hedgehog on state, MAPK6/MAPK4 signaling, Mitotic Anaphase, Cell Cycle, Mitotic, p53-Dependent G1 DNA Damage Response, Cell Cycle Checkpoints, Gene expression (Transcription), Signaling by PTK6, Metabolism of RNA, Fc epsilon receptor (FCERI) signaling, and, Ubiquitin-like domain superfamily. Psmf1(proteasome inhibitor subunit 1) is interlinking with four lncRNAs, i.e., ubc, gli3, femt2, oaz1a with the functions of Protein-containing complex binding, PIP3 activates AKT signaling, SCF(Skp2)-mediated degradation of p27/p21, Cellular responses to stress, PCP/CE pathway, GLI3 is processed to GLI3R by the proteasome, Hedgehog on state, MAPK6/MAPK4 signaling, Mitotic Anaphase, Cell Cycle, Mitotic, p53-Dependent G1 DNA Damage Response, Cell Cycle Checkpoints, Gene expression (Transcription), and, Metabolism of RNA. Egr1 (early growth response 1) is linked with fosab and ptenb lncRNA with the function of Cellular responses to stress, Cellular Senescence, and Fc epsilon receptor (FCERI) signaling. RANBP2 (RNA binding protein 2) is interlinking with tpm4a, srrm1, ndc1with the function of RNA transport, Cellular responses to stress, Mitotic Anaphase, Cell Cycle, Mitotic, Cell Cycle Checkpoints, Gene expression (Transcription), Metabolism of RNA, Transport of the SLBP independent and dependent Mature mRNA, Nuclear Pore Complex (NPC) Disassembly, Transport of Mature mRNA Derived from an Intronless Transcript, SUMOylation of SUMOylation proteins, SUMOylation of chromatin organization proteins, SUMOylation of RNA binding proteins and DNA replication proteins. Of the 35 lncRNAs, RANBP2 is the most significantly interlinked with ndc1 and egr1, with fossil lncRNA data enclosed in (S2 Table in S1 File).

## Discussion

In this study, the water and sediment had pH values between 7.60 and 8.3, indicating the alkaline character of the environment [19]. Conductivity is an indicator of the overall ionic content of water and, hence, of its freshness. Water’s conductivity is influenced by temperature, dissolved particle fluctuations, and ionic content (Ca2^ + ^, Mg2^ + ^, HCO3^-^, CO3^-^, NO_3_^-,^ and PO_4_^-^) [20]. The conductivity values of non-polluted and polluted water samples were 375 and 633 µ S/cm, which is the standardized value of water according to USPH (APHA). Another study shows that the conductivity of water varies between 50-1500 µ S/cm. River water is considered fresh since it contains less than 0.5 parts per thousand of salt [21]. In this study, the non-polluted and polluted water salinity was 0.40 and 0.29 ppm. DO, which denotes physical and biological processes affecting the water system, is one of the most crucial factors in assessing water quality. Non-polluted water has 5.60 ppm DO, compared to 4.5 ppm in polluted water. The lower value of DO shows the affected water bodies. The standard ideal amount of DO in water is determined to be 4-6 mg L^-1^ [22]. The BOD and COD values of non-polluted and polluted water samples were 1.4 ppm, 7.6 ppm, and 5.0 ppm, 8.0 ppm, higher than the standardized value of 4.0 ppm. According to APHA, the river’s lower BOD and greater COD levels suggest dumping low-volatile matter-containing waste. Sediment conductivity of non-polluted and polluted sites were 254 μS/cm, 866 μS/cm. The quality of sediment is not suitable due to the absorption of waste materials and the many activities caused by humans, such as the wading of cattle, the discharge of wastewater, the discharge from factories and tanneries, and the dumping of ceremonial garbage. In Yamuna River, chloride and fluoride were in high percentages [9]. A consistent increase in the levels of conductivity, biological oxygen demand (BOD), and chemical oxygen demand (COD) in the water and sediment of the Yamuna River was found to be the result of the direct discharge of untreated chemicals, ternary waste, and other types of industrial waste.

Due to riverine water pollution, the data showed that 6206 genes were downregulated and 2538 genes were upregulated. When comparing the low and high salinity groups, a study found 128 significant DEGs in the former, 43 of which were up-regulated and 85 of which were down-regulated in response to salt [23]. In *Rita rita*, from 2024, DEGs were found using DESeq; these genes included 942 upregulated genes and 1082 down-regulated genes compared to the non-polluted stretches [16]. The top 20 BP, MF, and CC of GO may serve in the right direction for the functional analysis of this species. Ribosome biogenesis, peptide biosynthetic process, peptide metabolic process, translation, cellular amide metabolic process, etc., in BP, structural constituents of the ribosome and RNA binding are most prevalent, and oxidoreductase activity in MF is significantly high. It was found that when *Larimichthys crocea* was under alkaline stress, the nicotinamide nucleotide metabolic process, the pyridine nucleotide metabolic process, and the pyridine-containing compound metabolic process were more active in the BP category [24]. Inside the MF group, the phospho-fructokinase activity, the carbohydrate kinase activity, and the neurotransmitter transporter activity all changed [25]. Cholesterol delta-isomerase, steroid metabolic, fatty acid biosynthetic, and sterol metabolic processes were highly regulated in *Cynoglossus semilaevis* at low salinity in biological processes and molecular functions [23]. A study observed that the river Ganga pollution primarily disrupted the ribosomal large subunit biogenesis and ribosome biogenesis groups in the hepatic transcriptome of *Rite Rita* due to biological processes [16]. In GO functional analysis, many genes are involved in ribosomal biogenesis of upregulated DEGs of Gynogenetic Mrigal carp liver tissue in cold stress [26].

KEGG provides an extensive database to efficiently investigate gene activities and create links between genomic data and comprehensive functional information [27]. In this study, metabolic pathways, 253 genes are linked with an FDR value of 2.264, while 64 genes are linked with ribosome pathways (RP) with a high FDR value of 7.767. In Gynogenetic Mrigal carp’s brain and liver tissue, the ribosome pathway was associated with many genes in KEGG analysis in response to acute cold stress [26]. Ribosomal protein L (rpl) genes are essential for the synthesis of the eukaryotic ribosome’s 60S component, and they may also undertake extra-ribosomal functions [28]. rps7e, rps10e, rps15e, rpl19, rps2e, and rpl23Ae, etc, are overexpressed in the ribosomal pathways in this study. Mutations in ribosomal proteins have been associated with zebrafish growth retardation and cancer susceptibility. Defects in development may occur via independent and p53-dependent pathways when RPS7 is differentially expressed [29]. According to a different study, Znf598’s ubiquitination of Rps10/eS10 contributes to establishing a characteristic ubiquitination pattern in zebrafish [30]. Also, Rps15e is differentially expressed in zebrafish on the effect of toxicity [31]. RPL19 belongs to a family of around 70 proteins that build large and small ribosomes. While exposed to copper, the RPL19gene becomes active in the liver of freshwater swordtail fish [32]. Mammals are the primary hosts for the cytoplasmic protein RPS2, which has a highly conserved sequence design. Through binding to the ribosome, aminoacyl-transfer RNA may impact the accuracy of mRNA translation. The 40S ribosomal subunit’s outer surface is where the S2 protein is located [33]. As a member of the 60S subunit, RPL23A is involved in ribosome-transfer RNA (tRNA) interaction processing. Protein synthesis is controlled by RPS7, which is found in the 40S subunit [34]. Ten RP genes with varying paralogous gene counts were discovered in tilapia in a separate study that aimed to decipher the RP gene’s evolutionary trajectory in vertebrates through phylogenetic and syntenic analyses. These genes include two each for rpl3, rpl5, rpl7, rpl19, rpl41, rpl2, rps17, and rps19; three from rpl22; and four from rps27.

Interestingly, this study found that FGG mRNA is co-expressed in up and down-regulation in the liver of *l. calbasu*. FGG is a fast-growing group of growth hormones. The expression of the GH gene in FGG was considerably more significant than that in SGG in the brain containing the pituitary gland of *Epinephelus akaara*. However, it was discovered that SGG had the most important levels of IGF1 and GHR gene expression in its muscles [35]. On the contrary, the mRNA expressions of Per1 and Per2 oscillate in threespot wrasse when subjected to persistent light and darkness. Furthermore, the retina and hypothalamus of trout exposed to complete darkness maintain the regular pattern of Per1 gene expression [36]. In our study, PER1 is up-regulated in response to pollution stress. In a few studies, mRNA expression levels were shown as upregulated. The researcher found that the gills of *O. mykiss* and *Misgurnus anguillicaudatus* both show an up-regulated pattern of C3 mRNA expression in response to biotic stress [37]. In our study, C3 mRNA was upregulated.

The findings of this study provide insights into the biological processes of pollution-stressed fish. lncRNA are silent conductors that direct the transcriptional ensemble with finesse and precision. More and more research points to lncRNA as a critical regulator that impacts cell proliferation, differentiation, metastasis, and death [38]. LncRNAs may regulate gene expression via cis- and trans-regulation to help preserve cellular equilibrium and operation after environmental stresses [39]. In various biological functions like reproduction, embryonic development, sex differentiation, immunity, and metabolism at the transcriptional, post-transcriptional, and epigenetic levels, involvement of LncRNAs interact with RNA, DNA, or proteins to affect target genes. LncRNAs in fish played a significant role in dealing with stress by the network regulation of gene expression [40].

Several lncRNA transcripts showed differential expression in *L. waleckii* gill and kidney tissues cultured in moderately and strongly alkaline conditions. While 6,571 lncRNAs were found in the kidneys, 5,244 were found in the gills. Findings from this study point to the potential metabolic function of lncRNAs in *L. waleckii* adaptation to extreme alkaline conditions [41]. lncRNAs are involved in various essential stress responses tilapia [42]. RANBP2 is the most significantly interlinked with Ndc1 and Egr1 (Early growth response 1), with fosab lncRNA. RANBP2 is primarily responsible for neuron plasticity and cancer cells, particularly in the glial cells, and dopaminergic cell toxicity induced by various neural toxic elements, either biological or environmental. The role of RNBP2 in aquatic environments and, subsequently, in fish is not well documented yet. However, the present findings in the transcriptome study taking *L. calbasu* as an experimental model that give an insight into the functional role of this gene in the neurotoxic pathways that needs further detailed study. Earlier research found that Egr1 is crucial to zebrafish retinal development. Egr1 was first found to be an early response gene that quickly changed in reaction to different growth factors and played a part in cell division, proliferation, and neural plasticity. The highly conserved zinc finger protein Egr1 is involved in several aspects of vertebrate development and adulthood. Many environmental challenges are known to stimulate Egr1, and treatment with these stresses is associated with an increase in DNA binding activity and protein phosphorylation. A transcription factor that controls cellular responses to stress, hypoxia, and environmental changes is encoded by the EGR1 gene. Ecotoxicological research focuses on EGR1 because it is essential to understanding how organisms adapt to pollution and other environmental stresses in aquatic habitats [43]. In the retina, egr1 serves a variety of roles [44]. A key component of the nuclear pore complex is RANBP2, which has a Small ubiquitin-related modifier (SUMO) E3-ligase domain, which changes several proteins post-translationally and controls mRNA metabolism [45]. To complete the process of sumoylation, energy and an enzymatic cascade are required. This cascade includes an E3 ligase, the SUMO-specific E1-activating enzyme Aos1/Uba2, and the E2-conjugating enzyme Ubc9. RanBP2/Nup358 protein is one of the few SUMO E3 ligases that have been discovered [46]. Recent studies have revealed that SUMOylation plays an important role in regulating stress granule (SG) assembly. The recruitment of several SUMO ligases, such as RANBP2 and UBE2I, by SGs has been established in recent proximity labeling studies [47]. Biotic stress induced eukaryotic translation initiation factor-3 subunit K (eIF3k) in a bacterial model reported by Chen et al. In our present research, we also notice the upregulation of elf3k, possibly due to stress factors associated with riverine pollution [48].

## Limitations

However, this study has substantial limitations, such as a small sample size, which may affect the generalizability of our findings across a broader ecosystem. The limited sampling locations may not be able to adequately represent the spatial variability of microbial communities and pollution impacts. Furthermore, our analysis must focus on all possible confounding factors, including age, gender, multimorbidity, and reproductive season, which may influence liver gene expression. Our objective for future research is to expand sampling to additional sites and to collect more samples (gill, muscle, and liver) that can be expanded to other directions of study to obtain clearer insights into the pollution impact on this specific species and incorporate multi-omics techniques, including proteomics, metabolomics, and transcriptomics, to gain a more comprehensive understanding of pollution effects on fish health.

## Conclusions

In conclusion, the research analyzed the hepatic transcriptome of *L. calbasu* to discover the potential genes and pathways influenced by pollution. The findings revealed a substantial number of genes that were expressed differentially. 6,206 DEGs were downregulated, and 2538 DEGs were upregulated out of 8744 DEGs. Important pathways and the genes involved were uncovered by GO and KEGG analyses. The metabolic and ribosome pathways were the most important in KEGG analysis, with high FDR values and many genes. Essential genes, including mgat2, ehhadh, pik3cb, pmvk, ptdss2, mthfd2, cox11, acsbg1, and fktn, are associated with the metabolic pathways. These genes are responsible for a variety of metabolic processes. When it comes to the pathway of the ribosome, the majority of genes, including rpl3, rps2e, rps10e, rpl23Ae, rpl19e, rps15e, and rps7e, amongst others, are exposed to higher levels of expression than they should be. In this regard, 35 lncRNAs are interlinked with each other. Ubc, Psmf1, RANBP2, egr1, and tmp4a are most significantly interlinked with other lncRNAs. FGG mRNA is co-expressed in both up and down-regulation, and the expression of the GH gene in FGG is considerably higher compared to SGG in the brain containing the pituitary gland. The results of our analysis provide essential resources that may be used to understand better the molecular pathways, important lncRNAs, and mRNAs that *L. calbasu* employs in response to riverine pollution. However, as some of the identified genes are not yet well documented, future research will focus on their functional characterization and regulatory roles. Leveraging multi-omics approaches will provide deeper insights into molecular processes. Understanding how metabolic and ribosomal genes adapt over time can help to identify crucial biomarkers for monitoring fish health and preserving aquatic ecosystems.

## Supporting information

S1 File **S1** Table. Summary of KEGG Pathways Featuring Corresponding Fold Enrichment, Involved Genes, and Enrichment Values to Highlight Key Pathways. **S2** Table. Comprehensive Overview of LncRNAs, Including Their Differential Expression Patterns, FDRand p-Values for Statistical Significance.
(DOCX)
