# Supplementary material for: Comparative transcriptome analysis of Labeo calbasu (Hamilton, 1822) from polluted and non-polluted rivers in India
Source: PLoS One. 2025 Apr 10;20(4):e0320358. doi: 10.1371/journal.pone.0320358 (PMC11984975; doi:10.1371/journal.pone.0320358)
Supplement: S1 File — S1 Table. Summary of KEGG Pathways Featuring Corresponding Fold Enrichment, Involved Genes, and Enrichment Values to Highlight Key Pathways. S2 Table. Comprehensive Overview of LncRNAs, Including Their Differential Expression Patterns, FDRand p-Values for Statistical Significance. (DOCX) [file pone.0320358.s001.docx]

**S1 Table.** Summary of KEGG Pathways Featuring Corresponding Fold Enrichment, Involved Genes, and Enrichment Values to Highlight Key Pathways.

| **Si. No** | **Enrichment FDR** | **nGenes** | **Pathway Genes** | **Fold Enrichment** | **Pathway** | **Genes** |
| --- | --- | --- | --- | --- | --- | --- |
| 1 | 1.86E-39 | 64 | 130 | 7.767950731 | Path:dre03010 Ribosome | rps9 rps5 rpl35 rps18 rpl24 rpl3 rpl10a rpl14 rpl27 rpl12 rpl23a rplp2 mrpl3 rps3 rpl21 rpl17 rps26 rps14 rpl9 rpl7 rpl10 rpl30 rplp1 rpl6 rps15a rpl23 rps12 rpl7a rpl4 rps15 rps3a rps27a rpl13 rpl8 rps19 rps7 rps25 rps17 rpsa rpl18a rpl28 rpl34 rps10 rps24 rpl36 rpl19 rps2 rps11 rps8a rps13 rpl11 mrpl24 rpl35a rps6 rpl18 rpl15 rpl32 rpl22 rpl13a rplp0 uba52 rpl31 mrps6 rps16 |
| 2 | 5.88E-34 | 253 | 1763 | 2.264321288 | Path:dre01100 Metabolic pathways | mgat2 ehhadh pik3cb pmvk ptdss2 hal mthfd2 cox11 acsbg1 fktn dmgdh dpys dgke oplah gch1 chdh chpfa npr2 enpp2 mthfs ganab kynu aldh1l1 pnpla2 acy1 odc1 adh5 ugdh fads2 hgd cad cat rrm1 gapdh paics gldc cth bhmt cs fah nqo1 uap1 upb1 stt3b mtmr14 adi1 asmtl dlat taldo1 kmt2e rpn1 gclc pfkfb1 hsd3b7 lpcat4 hsd11b2 aldh7a1 adsl man1a2 rpn2 hadhb hagh hlcs kmo dlst ivd echs1 gys2 plcg1 tktb pck1 mtr pah eno3 abat scd agps idh2 smox ahcy lipg hsd17b4 sepsecs xylb etnppl pdhx selenbp1 ndufa8 prdx6 ndufb8 mtmr3 gne sdha fh aacs pgm1 sardh asns acadl qars1 sts gcat nit2 amdhd2 alg2 rgn mao hoga1 ethe1 hprt1 acadm acaa2 got1 uqcrfs1 gapdhs mdh2 ddost acox3 pck2 tdh amy2a lclat1 fggy nnt fpgs pgd cyp51 acaa1 rdh12 pnpla3 galm aldh6a1 ldhbb gstz1 gstk1 coq3 khk acss2 blvrb hadh auh dpm1 ndufv1 alg6 aldh8a1 sc5d aspdh DHDH galca acox1 amt pcyt2 atp6v0e1 mogat2 dera pgm3 csad haao ndufs1 tm7sf2 ephx2 lancl1 st3gal4 ndufa5 asrgl1 arsa acss1 dcxr nsdhl afmid ndufs3 esd sqor fdps pcxa pisd tusc3 ndufs2 gda ugcg bckdha gnpnat1 pfkfb3 btd plod3 aass acacb cyp2u1 setd1a uroc1 acmsd aldh18a1 tkta lpin2 pipox fasn gba2 acaca dot1l man2a1 dglucy xdh naga kmt2ca kmt2ba mtm1 tat kl nadkb ash1l glsa aldh5a1 dgat2 pld2 man1a1 suox uqcrh l2hgdh mthfr dtymk gfpt1 mogs kmt2cb aco1 h6pd mut bckdhb arg1 g6pd sord glyctk pank2 pik3c2a gusb miox fdft1 ppat oat acadvl gart agxt2 impad1 gck agpat5 amd1 cyp21a2 mocs1 tkfc fmo5 lss ndufb5 sirt1 galnt16 |
| 3 | 3.54E-21 | 57 | 197 | 4.56539617 | Path:dre04141 Protein processing in endoplasmic reticulum | march6 sec31b ganab xbp1 ube4b sec31a hsp90ab1 pdia6 stt3b plaa calr rpn1 ssr4 hyou1 vcp sec23b nploc4 ubqln4 syvn1 man1a2 rpn2 hspbp1 bcap31 ssr1 hspa5 pdia3 hsp90b1 dnajb11 skp1 prkcsh edem1 txndc5 ddost atf4a p4hb canx sec63 ube2g2 uggt1 dnajc1 sec24d tusc3 pdia4 map3k5 lman2 atf6 eif2ak3 lman1 ddit3 sec62 hsp90aa1.2 herpud1 man1a1 mogs sel1l sec24c selenos |
| 4 | 3.90E-14 | 34 | 106 | 5.06107639 | Path:dre03013 Nucleocytoplasmic transport | magoh kpna6 nup214 ddx19 nup93 eef1a1l1 ran ipo7 thoc2 kpna3 aaas nup155 ddx39b eif4a3 casc3 srrm1 upf1 nup133 xpo7 pnn ipo11 nup205 phax tnpo2 rbm8a ipo4 senp2 xpot nup188 nup107 kpnb3 kpnb1 nup153 nup98 |
| 5 | 3.09E-11 | 34 | 131 | 4.095222117 | Path:dre01200 Carbon metabolism | adh5 cat gapdh gldc cs dlat taldo1 dlst echs1 tktb eno3 idh2 sdha fh rgn got1 mdh2 acox3 pgd aldh6a1 acss2 acox1 amt acss1 esd pcxa tkta aco1 h6pd mut g6pd glyctk gck tkfc |
| 6 | 4.38E-11 | 29 | 99 | 4.622028765 | Path:dre04146 Peroxisome | pmvk cat sod1 pex19 pex26 sod2 agps idh2 hsd17b4 pex5 mpv17 hacl1 acox3 decr2 acaa1 gstk1 eci2 acox1 ephx2 prdx1 pecr phyh crot pipox xdh abcd1 pex11b pex6 pex1 |
| 7 | 3.27E-08 | 24 | 91 | 4.161402177 | Path:dre03320 PPAR signaling pathway | sorbs1 cpt2 acsbg1 plin1 apoa1b fads2 fabp3 lpl apoa1a aqp7 pck1 scd dbi acadl acadm acox3 pck2 acaa1 acox1 cpt1b pparg slc27a6 ubc plin2 |
| 8 | 5.42E-08 | 21 | 73 | 4.539063676 | Path:dre03008 Ribosome biogenesis in eukaryotes | CABZ01085878.1 ran wdr43 wdr3 wdr36 gnl3 xrn2 gtpbp4 heatr1 eif6 nat10 gar1 sbds nop56 fbl rpp40 rcl1 nop58 mdn1 rexo1 dkc1 |
| 9 | 6.60E-08 | 18 | 55 | 5.163921792 | Path:dre00071 Fatty acid degradation | ehhadh cpt2 acsbg1 adh5 aldh7a1 hadhb echs1 acadl acadm acaa2 acox3 acaa1 eci2 hadh acox1 cpt1b cyp2u1 acadvl |
| 10 | 2.18E-07 | 48 | 314 | 2.412022918 | Path:dre05132 Salmonella infection | snx33 pik3cb rilp elmo2 stx10 ctnnb1 hsp90ab1 gapdh arpc2 jun rps3 cyfip1 cdc42 kpna3 pak1 birc2 hsp90b1 skp1 klc1a dctn2 tab1 myd88 actr3 acbd3 tnfrsf1a rela txn fhod1 ikbkg dctn4 casp7 ptprc ahnak exoc4 nckap1 fadd flnb plekhm2 hsp90aa1.2 nfkb1 vps11 irak1 pik3c2a prx tuba1a casp8 map2k6 rab9a |
| 11 | 2.56E-07 | 13 | 31 | 6.616853193 | Path:dre00640 Propanoate metabolism | echs1 abat acox3 aldh6a1 ldhbb acss2 acox1 acss1 bckdha acacb acaca mut bckdhb |
| 12 | 8.52E-07 | 17 | 58 | 4.624776701 | Path:dre00280 Valine leucine and isoleucine degradation | ehhadh aldh7a1 hadhb ivd echs1 abat aacs acadm acaa2 acaa1 aldh6a1 hadh auh bckdha mut bckdhb agxt2 |
| 13 | 9.13E-07 | 28 | 143 | 3.089525859 | Path:dre03040 Spliceosome | magoh snw1 ddx5 prpf6 thoc2 xab2 tra2a eftud2 ddx39b crnkl1 prpf8 eif4a3 snrpb prpf3 hnrnpk sf3b3 prpf18 sart1 syf2 sf3b1 slu7 bcas2 rbm8a hnrnpm u2surp sf3b2 dhx8 sf3a1 |
| 14 | 9.13E-07 | 30 | 160 | 2.95849686 | Path:dre04120 Ubiquitin mediated proteolysis | herc3 keap1b map3k1 wwp1 ube4b cdc27 mdm2 ubr5 syvn1 cul2 herc1 rps27a birc2 xiap ube3c ddb1 skp1 uba1 ube2z ube2g2 herc2 huwe1 nedd4l wwp2 trip12 fbxw7 anapc1 uba52 ubc birc6 |
| 15 | 1.90E-06 | 32 | 184 | 2.74411303 | Path:dre05168 Herpes simplex virus 1 infection | pik3cb ppp1cb tyk2 bcl2l1 jak1 b2m tp53 mtor calr birc2 pdia3 eif2b3 tab1 myd88 syk tnfrsf1a rela ikbkg cfp pik3r1 eif2ak3 fadd eif2b5 stat2 nfkb1 c5 tsc2 irak1 casp8 apaf1 tbk1 |
| 16 | 3.20E-06 | 10 | 22 | 7.172113601 | Path:dre03060 Protein export | srp72 hspa5 srp54 spcs3 srp19 srprb sec63 srp68 spcs2 sec62 |
| 17 | 6.46E-06 | 21 | 98 | 3.381139269 | Path:dre03015 mRNA surveillance pathway | ppp1cb magoh ddx19 pabpc4 tardbp ddx39b eif4a3 casc3 ppp2r2d pabpn1 srrm1 upf1 pnn gspt1 wdr33 pcf11 rbm8a smg5 smg7 smg1 |
| 18 | 7.99E-06 | 18 | 76 | 3.737048666 | Path:dre01212 Fatty acid metabolism | cpt2 acsbg1 fads2 hadhb echs1 scd hsd17b4 acadl acadm acaa2 acox3 acaa1 hadh acox1 cpt1b fasn acaca acadvl |
| 19 | 1.00E-05 | 12 | 36 | 5.259549974 | Path:dre00630 Glyoxylate and dicarboxylate metabolism | cat gldc cs hoga1 mdh2 acss2 amt acss1 afmid aco1 mut glyctk |
| 20 | 2.83E-05 | 13 | 46 | 4.459183673 | Path:dre00260 Glycine serine and threonine metabolism | chdh gldc cth bhmt aldh7a1 sardh gcat mao tdh amt pipox glyctk agxt2 |
| 21 | 3.37E-05 | 11 | 34 | 5.104857328 | Path:dre00020 Citrate cycle (TCA cycle) | cs dlat dlst pck1 idh2 sdha fh mdh2 pck2 pcxa aco1 |
| 22 | 3.37E-05 | 14 | 54 | 4.090761091 | Path:dre00620 Pyruvate metabolism | adh5 dlat aldh7a1 hagh pck1 fh mdh2 pck2 ldhbb acss2 acss1 pcxa acacb acaca |
| 23 | 3.37E-05 | 19 | 93 | 3.223595145 | Path:dre04137 Mitophagy-animal | bcl2l1 tp53 jun bnip3 optn rps27a sqstm1 atf4a rela fundc1 pgam5 calcoco2 tbc1d17 eif2ak3 usp8 mfn2 uba52 tbk1 ubc |
| 24 | 5.77E-05 | 11 | 36 | 4.821254143 | Path:dre00410 beta-Alanine metabolism | ehhadh dpys upb1 aldh7a1 echs1 abat smox acox3 aldh6a1 acox1 csad |
| 25 | 8.81E-05 | 35 | 255 | 2.165697048 | Path:dre04510 Focal adhesion | pik3cb ppp1cb mylk5 met rock1 itga4 igf1 lamc1 ctnnb1 rap1b shc1 col1a1b jun col1a2 cdc42 col1a1a pak1 birc2 xiap erbb2 itga9 pak4 crk ppp1r12a tln1 flt1 kdr col4a1 pik3r1 flnb itgb5 col6a2 ppp1r12c arhgap5 |
| 26 | 8.94E-05 | 29 | 194 | 2.358664164 | Path:dre04210 Apoptosis | pik3cb bcl2l1 lmna tp53 jun ctsc birc2 xiap aifm1 tnfrsf1a atf4a rela ctsz ikbkg sptan1 casp7 map3k5 pik3r1 parp4 eif2ak3 ptpn13 ddit3 fadd ctsf nfkb1 tuba1a casp8 apaf1 ctsd |
| 27 | 9.15E-05 | 13 | 52 | 3.94466248 | Path:dre00380 Tryptophan metabolism | ehhadh kynu cat aldh7a1 kmo dlst echs1 mao hadh aldh8a1 haao afmid acmsd |
| 28 | 0.000117141 | 12 | 46 | 4.116169545 | Path:dre00250 Alanine aspartate and glutamate metabolism | cad adsl abat asns nit2 got1 asrgl1 glsa aldh5a1 gfpt1 ppat agxt2 |
| 29 | 0.000128396 | 10 | 33 | 4.781409067 | Path:dre00030 Pentose phosphate pathway | taldo1 tktb pgm1 rgn pgd dera tkta h6pd g6pd glyctk |
| 30 | 0.000128396 | 17 | 86 | 3.11903545 | Path:dre01230 Biosynthesis of amino acids | acy1 gapdh cth cs taldo1 tktb mtr pah eno3 idh2 asns got1 pcxa aldh18a1 tkta aco1 arg1 |
| 31 | 0.000206767 | 40 | 323 | 1.954012374 | Path:dre04144 Endocytosis | arfgap1 arfgef1 wwp1 prkci mdm2 ist1 agap3 arpc2 hgs ap2b1 capzb cdc42 fgfr2 stam chmp7 amph dab2 chmp2bb actr3 clta pdcd6ip epn1 spg21 rab11a eea1 igf2r psd2 ap2a1 nedd4l zfyve27 vps35 ehd3 usp8 pld2 gbf1 rabep1 arfgap2 asap3 eps15 chmp2a |
| 32 | 0.00021814 | 25 | 165 | 2.390704534 | Path:dre04621 NOD-like receptor signaling pathway | tyk2 bcl2l1 trpm7 jak1 hsp90ab1 vdac2 jun birc2 xiap tab1 myd88 rela txn ikbkg atg16l1 fadd tnfaip3 hsp90aa1.2 stat2 nfkb1 erbin mfn2 casp8 tbk1 sharpin |
| 33 | 0.000266259 | 11 | 43 | 4.036398817 | Path:dre00970 Aminoacyl-tRNA biosynthesis | kars1 dars1 aars1 iars1 rars1 gars1 sepsecs qars1 tars1 farsb nars1 |
| 34 | 0.000305772 | 13 | 59 | 3.476651678 | Path:dre00510 N-Glycan biosynthesis | ganab stt3b rpn1 man1a2 rpn2 alg2 ddost dpm1 alg6 tusc3 man2a1 man1a1 mogs |
| 35 | 0.000621389 | 12 | 55 | 3.442614528 | Path:dre00270 Cysteine and methionine metabolism | cth bhmt adi1 gclc mtr ahcy got1 mdh2 ldhbb tat agxt2 amd1 |
| 36 | 0.00067517 | 15 | 81 | 2.921972208 | Path:dre00310 Lysine degradation | ehhadh kmt2e aldh7a1 dlst echs1 hadh plod3 aass setd1a pipox dot1l kmt2ca kmt2ba ash1l kmt2cb |
| 37 | 0.00198445 | 13 | 71 | 2.889048577 | Path:dre00561 Glycerolipid metabolism | dgke pnpla2 lpl aldh7a1 lipg lclat1 pnpla3 mogat2 lpin2 dgat2 glyctk agpat5 tkfc |
| 38 | 0.002166484 | 14 | 81 | 2.727174061 | Path:dre00010 Glycolysis/Gluconeogenesis | adh5 gapdh dlat aldh7a1 pck1 eno3 pgm1 gapdhs pck2 galm ldhbb acss2 acss1 gck |
| 39 | 0.002166484 | 16 | 100 | 2.524583987 | Path:dre04620 Toll-like receptor signaling pathway | pik3cb cd40 jun tollip tab1 myd88 rela ikbkg pik3r1 fadd map3k8 nfkb1 irak1 casp8 map2k6 tbk1 |
| 40 | 0.00310406 | 24 | 187 | 2.02506737 | Path:dre04140 Autophagy-animal | pik3cb zfyve1 ulk2 rb1cc1 bcl2l1 mtmr14 mtor bnip3 ddit4 mtmr3 sqstm1 igbp1 atg16l1 pik3r1 dapk1 eif2ak3 rptor dapk3 pik3r4 akt1s1 tsc2 ctsd tbk1 deptor |
| 41 | 0.004406193 | 6 | 20 | 4.733594976 | Path:dre00670 One carbon pool by folate | mthfs aldh1l1 mtr amt mthfr gart |
| 42 | 0.004782983 | 9 | 43 | 3.302508123 | Path:dre01250 Biosynthesis of nucleotide sugars | ugdh uap1 gne pgm1 pgm3 gnpnat1 gfpt1 miox gck |
| 43 | 0.005263967 | 22 | 173 | 2.006533516 | Path:dre04910 Insulin signaling pathway | pik3cb sorbs1 ppp1cb prkci shc1 mtor gys2 pck1 pck2 rps6 crk ppargc1a acacb pik3r1 fasn acaca rptor tsc2 phkb gck srebf1 |
| 44 | 0.007085352 | 6 | 22 | 4.30326816 | Path:dre00100 Steroid biosynthesis | cyp51 sc5d tm7sf2 nsdhl fdft1 lss |
| 45 | 0.008645938 | 4 | 10 | 6.311459969 | Path:dre00920 Sulfur metabolism | ethe1 sqor suox impad1 |
| 46 | 0.008682685 | 6 | 23 | 4.116169545 | Path:dre00650 Butanoate metabolism | echs1 abat aacs hadh aldh5a1 l2hgdh |
| 47 | 0.009560218 | 9 | 48 | 2.95849686 | Path:dre00513 Various types of N-glycan biosynthesis | stt3b rpn1 man1a2 rpn2 alg2 ddost tusc3 man2a1 man1a1 |
| 48 | 0.012067028 | 21 | 175 | 1.893437991 | Path:dre04142 Lysosome | dnase2 psap npc2 ap1g1 ctsc clta lgmn galca ctsz arsa man2b1 npc1 igf2r naga ap1b1 ap3d1 ctsf gusb WDR7 ctsd tpp1 |
| 49 | 0.012610314 | 13 | 89 | 2.304746618 | Path:dre04920 Adipocytokine signaling pathway | acsbg1 stat3 mtor pck1 tnfrsf1a pck2 rela cpt1b ikbkg ppargc1a acacb adipor2 nfkb1 |
| 50 | 0.014307375 | 39 | 397 | 1.550043695 | Path:dre04010 MAPK signaling pathway | met map3k1 igf1 tp53 tek rap1b map3k4 jun cdc42 fgfr2 pak1 erbb2 dusp8a tab1 myd88 dusp1 tnfrsf1a atf4a rela nfkb2 nr4a1 mapkapk5 crk ikbkg ppp5c flt1 kdr map3k5 rapgef2 ddit3 flnb map3k8 jund nfkb1 nfatc1 irak1 fgf4 map2k6 relb |
| 51 | 0.01479957 | 7 | 34 | 3.248545572 | Path:dre01040 Biosynthesis of unsaturated fatty acids | fads2 scd hsd17b4 acox3 acaa1 acot7 acox1 |
| 52 | 0.015318053 | 8 | 43 | 2.935562776 | Path:dre00040 Pentose and glucuronate interconversions | ugdh xylb fggy DHDH dcxr kl sord gusb |
| 53 | 0.017368686 | 8 | 44 | 2.86884544 | Path:dre04623 Cytosolic DNA-sensing pathway | polr3e rela ikbkg polr3b nfkb1 polr3a adar tbk1 |
| 54 | 0.020534695 | 14 | 106 | 2.083972631 | Path:dre04012 ErbB signaling pathway | abl1 pik3cb cdkn1a shc1 mtor jun pak1 plcg1 erbb2 gab1 pak4 crk stat5b pik3r1 |
| 55 | 0.020791636 | 3 | 7 | 6.762278538 | Path:dre00400 Phenylalanine tyrosine and tryptophan biosynthesis | pah got1 tat |
| 56 | 0.020791636 | 21 | 186 | 1.781460475 | Path:dre01240 Biosynthesis of cofactors | mthfd2 gch1 kynu ugdh cad nqo1 gclc adsl kmo rgn ggh fpgs rdh12 coq3 aspdh haao afmid nadkb pank2 gusb mocs1 |
| 57 | 0.021111738 | 14 | 107 | 2.064496251 | Path:dre00564 Glycerophospholipid metabolism | ptdss2 dgke lpcat4 etnppl lclat1 pcyt2 gpcpd1 pisd lpcat3 lpin2 pnpla6 lpgat1 pld2 agpat5 |
| 58 | 0.022567948 | 10 | 66 | 2.390704534 | Path:dre00330 Arginine and proline metabolism | odc1 aldh7a1 smox mao hoga1 got1 aldh18a1 arg1 oat amd1 |
| 59 | 0.023431078 | 5 | 21 | 3.75682141 | Path:dre01210 2-Oxocarboxylic acid metabolism | acy1 cs idh2 got1 aco1 |
| 60 | 0.023672109 | 7 | 38 | 2.906593407 | Path:dre00350 Tyrosine metabolism | adh5 hgd fah mao got1 gstz1 tat |
| 61 | 0.023719033 | 10 | 67 | 2.355022376 | Path:dre00480 Glutathione metabolism | oplah odc1 rrm1 gclc idh2 prdx6 pgd gstk1 lancl1 g6pd |
| 62 | 0.023719033 | 21 | 190 | 1.743956044 | Path:dre04217 Necroptosis | tyk2 trpm7 jak1 hsp90ab1 stat3 vdac2 birc2 xiap aifm1 chmp7 chmp2bb sqstm1 tnfrsf1a stat5b pgam5 fadd tnfaip3 hsp90aa1.2 stat2 casp8 sharpin |
| 63 | 0.024706396 | 21 | 191 | 1.734825384 | Path:dre04218 Cellular senescence | pik3cb ppp1cb cdkn1a trpm7 tp53 mdm2 rbbp4 vdac2 mtor zfp36l2 rad1 sqstm1 rela pik3r1 hipk2 nfkb1 tsc2 nfatc1 map2k6 sirt1 |
| 64 | 0.03361265 | 15 | 126 | 1.878410705 | Path:dre04625 C-type lectin receptor signaling pathway | pik3cb mdm2 jun pak1 syk rela nfkb2 ikbkg pik3r1 stat2 bcl3 nfkb1 nfatc1 casp8 relb |
| 65 | 0.033890991 | 6 | 32 | 2.95849686 | Path:dre04136 Autophagy-other | ulk2 mtor igbp1 atg16l1 rptor pik3r4 |
| 66 | 0.034234278 | 12 | 93 | 2.035954829 | Path:dre04512 ECM-receptor interaction | itga4 lamc1 col1a1b col1a2 col1a1a itga9 col4a1 itgb5 agrn hspg2 col6a2 sdc4 |
| 67 | 0.036539604 | 4 | 16 | 3.94466248 | Path:dre00360 Phenylalanine metabolism | pah mao got1 tat |
| 68 | 0.036539604 | 28 | 287 | 1.53938048 | Path:dre04810 Regulation of actin cytoskeleton | pik3cb ppp1cb mylk5 rock1 itga4 f2 arpc2 cyfip1 cdc42 fgfr2 pak1 itga9 wasf2 actr3 pak4 limk2 crk ppp1r12a myh10 diaph2 pik3r1 ssh2b nckap1 iqgap2 itgb5 ppp1r12c fgf4 |
| 69 | 0.040934274 | 23 | 227 | 1.598717833 | Path:dre04530 Tight junction | cgnb rock1 map3k1 dlg1 prkci arpc2 rapgef6 jun cdc42 scrib erbb2 ppp2r2d actr3 myh10 map3k5 rapgef2 nedd4l arhgef2 dlg3 tuba1a llgl1 llgl2 myl6 |
| 70 | 0.040963967 | 11 | 85 | 2.041942931 | Path:dre04115 p53 signaling pathway | cdkn1a bcl2l1 igf1 ccng1 tp53 mdm2 mdm4 tsc2 siva1 casp8 apaf1 |
| 71 | 0.041362451 | 6 | 34 | 2.784467633 | Path:dre03020 RNA polymerase | polr2b polr1a polr3e polr2a polr3b polr3a |
| 72 | 0.041543396 | 9 | 64 | 2.218872645 | Path:dre00520 Amino sugar and nucleotide sugar metabolism | ugdh uap1 gne pgm1 amdhd2 pgm3 gnpnat1 gfpt1 gck |
| 73 | 0.046987849 | 8 | 55 | 2.295076352 | Path:dre03050 Proteasome | psmb6 psme3 psmc6 psmd12 psma4 psmc2 psmb2 psmb4 |
| 74 | 0.060589772 | 3 | 11 | 4.30326816 | Path:dre00130 Ubiquinone and other terpenoid-quinone biosynthesis | nqo1 coq3 tat |
| 75 | 0.067374766 | 9 | 70 | 2.028683561 | Path:dre03250 Viral life cycle-HIV-1 | ran cdk9 ell hgs pdcd6ip samhd1 aff4 nup153 supt5h |
| 76 | 0.077863537 | 9 | 72 | 1.97233124 | Path:dre04330 Notch signaling pathway | jag1b dlc dld psen1 her2 snw1 ncor2 notch2 notch3 |
| 77 | 0.089005095 | 5 | 31 | 2.544943536 | Path:dre00790 Folate biosynthesis | gch1 pah ggh fpgs mocs1 |
| 78 | 0.09009721 | 8 | 63 | 2.003638085 | Path:dre04622 RIG-I-like receptor signaling pathway | map3k1 rela ikbkg fadd nfkb1 ddx3xa casp8 tbk1 |
| 79 | 0.092423861 | 4 | 22 | 2.86884544 | Path:dre00592 alpha-Linolenic acid metabolism | fads2 acox3 acaa1 acox1 |
| 80 | 0.092423861 | 4 | 22 | 2.86884544 | Path:dre00770 Pantothenate and CoA biosynthesis | dpys upb1 csad pank2 |
| 81 | 0.105500486 | 17 | 176 | 1.52407414 | Path:dre04068 FoxO signaling pathway | pik3cb csnk1e cdkn1a igf1 cat mdm2 stat3 prmt1 sgk1 sod2 bnip3 pck1 pck2 usp7 pik3r1 foxo4 sirt1 |
| 82 | 0.105500486 | 12 | 113 | 1.675608841 | Path:dre04520 Adherens junction | sorbs1 ptprb met cdh1 ctnnb1 ctnna1 cdc42 erbb2 wasf2 clul1 lmo7a ctnnd1 |
| 83 | 0.109192953 | 6 | 44 | 2.15163408 | Path:dre00053 Ascorbate and aldarate metabolism | ugdh aldh7a1 rgn kl gusb miox |
| 84 | 0.11556181 | 4 | 24 | 2.629774987 | Path:dre00511 Other glycan degradation | man2b1 gba2 engase man2c1 |
| 85 | 0.11556181 | 7 | 56 | 1.97233124 | Path:dre04216 Ferroptosis | tp53 vdac2 gclc ncoa4 lpcat3 slc40a1 slc39a14 |
| 86 | 0.128172704 | 4 | 25 | 2.524583987 | Path:dre00340 Histidine metabolism | hal aldh7a1 mao uroc1 |
| 87 | 0.14682993 | 6 | 48 | 1.97233124 | Path:dre02010 ABC transporters | abcc3 abcc5 abcc4 abcc2 abcd1 abcc12 |
| 88 | 0.149761995 | 9 | 84 | 1.690569634 | Path:dre03018 RNA degradation | patl1 hspd1 pabpc4 xrn2 hspa9 eno3 exosc10 cnot1 edc4 |
| 89 | 0.149761995 | 18 | 200 | 1.420078493 | Path:dre04150 mTOR signaling pathway | pik3cb ulk2 igf1 sgk1 mtor fnip1 ddit4 tnfrsf1a rps6 pik3r1 lpin2 rptor depdc5 akt1s1 tsc2 tti1 flcn deptor |
| 90 | 0.17111721 | 4 | 28 | 2.254092846 | Path:dre00220 Arginine biosynthesis | acy1 got1 glsa arg1 |
| 91 | 0.175697899 | 3 | 18 | 2.629774987 | Path:dre00450 Selenocompound metabolism | cth mtr sepsecs |
| 92 | 0.196638491 | 5 | 41 | 1.9242256 | Path:dre00062 Fatty acid elongation | hadhb echs1 acaa2 hadh acot7 |
| 93 | 0.196638491 | 13 | 142 | 1.444524289 | Path:dre00190 Oxidative phosphorylation | cox11 ndufa8 ndufb8 sdha uqcrfs1 ndufv1 atp6v0e1 ndufs1 ndufa5 ndufs3 ndufs2 uqcrh ndufb5 |
| 94 | Enrichment FDR | nGenes | Pathway Genes | Fold Enrichment | Pathway | Genes |

**S2 Table**. Comprehensive Overview of LncRNAs, Including Their Differential Expression Patterns, FDRand p-Values for Statistical Significance.

| **Si. No.** | **Lnc RNAs** | **Description** | **FDR value** | **p-value** |
| --- | --- | --- | --- | --- |
| 1 | ubtd1a\|tmub1\|ubtd1b\|ubc | Ubiquitin homologues | 4.50E-04 | 5.62E-07 |
| 2 | ndc1\|rpp40\|phax\|srrm1\|RANBP2 | RNA transport | 4.90E-04 | 2.96E-06 |
| 3 | ubtd1a\|tmub1\|ubtd1b | Mixed, incl. Ubiquitin-binding domain, and Forebrain neuron fate commitment | 9.10E-04 | 1.66E-07 |
| 4 | ubtd1a\|tmub1\|ubtd1b\|ubc | Ubiquitin-like domain | 0.0031 | 5.62E-07 |
| 5 | tpm4a\|fermt2\|phax\|cdkn1bb\|eif3k\|xirp2a\|tpm3\|psmf1\|naa25 | Protein-containing complex binding | 0.0225 | 8.30E-06 |
| 6 | ubtd1a\|ubtd1b | Viral transcriptional complex | 0.0257 | 1.63E-05 |
| 7 | cdkn1bb\|rac1a\|psmf1\|ubc | PIP3 activates AKT signaling | 0.0307 | 2.60E-04 |
| 8 | cdkn1bb\|psmf1\|ubc | SCF(Skp2)-mediated degradation of p27/p21 | 0.0307 | 5.85E-05 |
| 9 | ndc1\|fosab\|cdkn1bb\|psmf1\|ubc\|RANBP2 | Cellular responses to stress | 0.0307 | 1.91E-05 |
| 10 | fosab\|cdkn1bb\|ubc | Cellular Senescence | 0.0307 | 4.40E-04 |
| 11 | rac1a\|psmf1\|ubc | PCP/CE pathway | 0.0307 | 1.80E-04 |
| 12 | gli3\|psmf1\|ubc | GLI3 is processed to GLI3R by the proteasome | 0.0307 | 5.26E-05 |
| 13 | gli3\|psmf1\|ubc | Hedgehog on state | 0.0307 | 1.30E-04 |
| 14 | rac1a\|psmf1\|ubc | MAPK6/MAPK4 signaling | 0.0307 | 1.20E-04 |
| 15 | ndc1\|psmf1\|ubc\|RANBP2 | Mitotic Anaphase | 0.0307 | 1.60E-04 |
| 16 | ndc1\|cdkn1bb\|psmf1\|ubc\|RANBP2 | Cell Cycle, Mitotic | 0.0307 | 2.70E-04 |
| 17 | cdkn1bb\|psmf1\|ubc | p53-Dependent G1 DNA Damage Response | 0.0307 | 6.47E-05 |
| 18 | cdkn1bb\|psmf1\|ubc\|RANBP2 | Cell Cycle Checkpoints | 0.0307 | 3.00E-04 |
| 19 | ndc1\|phax\|cdkn1bb\|psmf1\|ubc\|RANBP2 | Gene expression (Transcription) | 0.0307 | 3.90E-04 |
| 20 | cdkn1bb\|rac1a\|ubc | Signaling by PTK6 | 0.0307 | 3.31E-05 |
| 21 | ndc1\|rpp40\|psmf1\|ubc\|RANBP2 | Metabolism of RNA | 0.0307 | 3.80E-04 |
| 22 | fosab\|rac1a\|ubc | Fc epsilon receptor (FCERI) signaling | 0.0323 | 5.90E-04 |
| 23 | ndc1\|RANBP2 | Transport of the SLBP independent Mature mRNA | 0.0337 | 6.50E-04 |
| 24 | ndc1\|RANBP2 | Transport of the SLBP Dependant Mature mRNA | 0.0337 | 6.90E-04 |
| 25 | ndc1\|RANBP2 | Transport of Mature mRNA Derived from an Intronless Transcript | 0.0337 | 6.50E-04 |
| 26 | ndc1\|RANBP2 | Nuclear Pore Complex (NPC) Disassembly | 0.0337 | 6.50E-04 |
| 27 | ndc1\|RANBP2 | SUMOylation of SUMOylation proteins | 0.0337 | 7.40E-04 |
| 28 | tpm4a\|eif3k\|xirp2a\|tpm3 | Cardiovascular system | 0.0366 | 0.0041 |
| 29 | ubtd1a\|ubtd1b | Ubiquitin-binding domain | 0.0392 | 1.63E-05 |
| 30 | tpm4a\|tpm3 | Tropomyosin | 0.0411 | 3.41E-05 |
| 31 | tpm4a\|tpm3 | Tropomyosin | 0.0452 | 3.41E-05 |
| 32 | ubtd1a\|tmub1\|ubtd1b\|ubc | Ubiquitin-like domain superfamily | 0.0452 | 3.95E-05 |
| 33 | ubtd1a\|ubtd1b | DC-UbP/UBTD2, N-terminal domain | 0.0452 | 1.63E-05 |
| 34 | ubtd1a\|ubtd1b | DC-UbP/UBTD2, N-terminal domain superfamily | 0.0452 | 1.63E-05 |
| 35 | ubtd1a\|ubtd1b | Ubiquitin domain-containing protein 1/2 | 0.0452 | 1.63E-05 |
| 36 | ndc1\|RANBP2 | SUMOylation of chromatin organization proteins | 0.0498 | 0.0012 |
| 37 | ndc1\|RANBP2 | SUMOylation of RNA binding proteins | 0.0498 | 0.0012 |
| 38 | ndc1\|RANBP2 | SUMOylation of DNA replication proteins | 0.0498 | 0.0012 |
